# Letrozole as premedication of high intensity focused ultrasound treatment of uterine fibroids: A retrospective observation study

**DOI:** 10.3389/fmed.2022.1069654

**Published:** 2022-12-06

**Authors:** Wei-Chun Chen, Chia-Chen Hsu, Huei-Jean Huang, Wei-Jen Cheng, Ting-Chang Chang, Hung-Hsueh Chou

**Affiliations:** ^1^Institute of Biomedical Engineering, National Tsing Hua University, Hsinchu, Taiwan; ^2^Division of Gynecologic Oncology, Department of Obstetrics and Gynecology, Chang Gung Memorial Hospital, Taoyuan, Taiwan; ^3^Department of Obstetrics and Gynecology, New Taipei City Municipal Tucheng Hospital, New Taipei City, Taiwan; ^4^High Intensity Focused Ultrasound (HIFU) Treatment Center, Chang Gung Memorial Hospital, Taoyuan, Taiwan; ^5^College of Medicine, Chang Gung University, Taoyuan, Taiwan; ^6^Graduate Institute of Clinical Medical Sciences, College of Medicine, Chang Gung University, Taoyuan, Taiwan; ^7^Center for Traditional Chinese Medicine, Chang Gung Memorial Hospital, Taoyuan, Taiwan; ^8^School of Traditional Chinese Medicine, Chang Gung University, Taoyuan, Taiwan; ^9^School of Medicine, National Tsing Hua University, Hsinchu, Taiwan

**Keywords:** letrozole, high intensity focused ultrasound, HIFU, uterine fibroid, gonadotrophin releasing hormone analog

## Abstract

**Background:**

No reports on Letrozole as a pretreatment before ablation of uterine fibroid with high intensity focused ultrasound (HIFU), so a retrospective observation study was performed to evaluate the response of different pre-HIFU medication.

**Methods:**

We collected patients with single uterine fibroid receiving HIFU ablation from January 2018 to April 2021. All enrolled patients were classified into three group: group A (no pre-HIFU medication use), group B (Pre-HIFU letrozole use), group C (pre-HIFU gonadotrophin releasing hormone analog, GnRHa). Further associated clinical data and treatment response after HIFU treatment were reviewed and evaluated.

**Results:**

A total of 39 patients including 21, 7, and 11 in group A, B, and C were collected respectively. After pre-HIFU medication, no difference of fibroid volume was found (A: 251.4, B: 360.6, C: 409.4 cm^3^, *p* = 0.250), and GnRHa group had significantly larger volume reduction than Letrozole users (38.6% vs. 16.4%, *p* = 0.007). The incidence of hypoestrogenic symptoms was higher in GnRHa group than in letrozole users (27.3% vs. 0, *p* = 0.170). GnRHa group had more sonication time (*p* = 0.001), treatment duration (*p* = 0.002), and ablated energy (*p* = 0.001) than group A and B. The treatment efficiency was higher in letrozole group than that in other 2 groups (4.52 vs. 2.39 vs. 2.34 cm^3^/min, *p* = 0.050). For patients with fibroid over 10 cm in diameter, letrozole group had even better energy efficiency (*p* = 0.067), treatment speed (*p* = 0.007), treatment efficiency (*p* = 0.001), NPV per energy (*p* = 0.005), and NPV per sonication (*p* = 0.004) than other 2 groups.

**Conclusion:**

Letrozole as a pretreatment medication before HIFU treatment might increase the energy efficiency and treatment efficiency of its ablation of uterine leiomyoma, especially for fibroid over 10 cm. Future study of larger patient number is needed to confirm our results.

## Introduction

Uterine leiomyomas are one of most prevalent benign gynecology diseases existing in more than 60% of women (1). Leiomyomas might cause symptoms including hypermenorrhea, resultant anemia, and bulky mass led to compression effects, such as abnormal defecation, dysfunctional voiding, abdominal protruding fullness, or even low back soreness (2). Hormonal treatment, like gonadotropin-releasing hormone agonist (GnRHa), ulipristal, and letrozole, had proved efficacy of volume-reduction (3). Previous literature also found the benefit and potential efficacy for Vitamin D and Myo-inositol for uterine fibroid management (4–7). Laparotomic or minimally-invasive myomectomy or hysterectomy are common operations currently (8).

Ablation of uterine leiomyomas with high-intensity focused ultrasound (HIFU) had been developed for years and has become an option of non-surgical treatment (9). Unlike medical diagnostic ultrasounds with acoustic intensity < 0.1 W/cm^2^, HIFU produces high intensity acoustic energy over 100 W/cm^2^ which can be focused into the targeted tumor to provide local heat over 60°C and generate local tissue ablation by accompanied cavitation and mechanic physical effects (10, 11). The leiomyoma after hyperthermia ablation may triggered coagulation necrosis in early phase and further cell apoptosis in late phase (12, 13). The treated leiomyoma cells may be engulfed or digested by immune or inflammatory cells, such as macrophage, and then the ablated leiomyomas shrink (14–16).

Combination of HIFU and hormonal treatment can be also an available therapy for uterine leiomyoma and adenomyosis. Hormonal treatment can be used before or after HIFU treatment to control the related menstrual symptoms of leiomyoma or adenomyosis, make HIFU treatment much more efficiently, or generate better tumor ablation or more volume reduction of targeted tumor after HIFU treatment (17). From previous literatures, GnRHa used before HIFU of leiomyomas or adenomyosis can make more efficient HIFU treatment with less consumption of energy (18, 19). Besides, GnRHa alone or combined with levonorgestrel intrauterine device can also be used after HIFU to make better volume reduction, prolonged symptoms relief, and improved quality of life (20, 21).

Letrozole is one of the third-generation aromatase inhibitors commonly used for hormonal treatment of breast cancer with expression of estrogen receptors (22). Uterine myometrium had abundant aromatase that can respectively converts androstenedione and testosterone to estrone and estradiol that furtherly trigger the development of leiomyoma stem cells (23–25). Letrozole decreases the circulating estrogen by inhibiting transformation of androgen into estrogen, and therefore have efficacy on leiomyoma treatment (26, 27). Previous research reported letrozole treatment before laparoscopic myomectomy can shorten the operation time and decrease the intraoperative blood loss (28). However, combining letrozole with HIFU had not been reported before. The current retrospective study was aimed at evaluating the clinical feasibility and efficacy of letrozole treatment before HIFU ablation of uterine leiomyoma.

## Materials and methods

### Patients and study design

We retrospectively reviewed all our patients with single leiomyoma receiving HIFU ablation at Chang Gung Memorial hospital during January 2018 until April 2021. This retrospective study was conducted with the approval of an institutional review board (IRB) (IRB Number 202200674B0). The associated clinical data including age at treatment, body mass index (BMI), the initial and following change of fibroid size based on ultrasound or magnetic resonance imaging (MRI), the pre-HIFU medication, post-HIFU surveillance, and other available information was obtained from electronic medical record system.

Every enrolled patient received MRI with contrast 1 month before and 1 day after HIFU ablation. The pre-HIFU checkup also included basic hemogram, liver function, kidney function, CA-125, LDH, cervical cytology smear, and gynecologic ultrasound. The major hormonal medication before HIFU ablation included GnRHa or letrozole based on physician's preference. All patients were classified into three group: group A as no pre-HIFU medication use, group B as pre-HIFU medication with letrozole, and group C as pre-HIFU medication with GnRHa. All of the patients had no hormonal therapy after HIFU treatment.

### Study drug: Letrozole and GnRHa treatment

In group B patients, letrozole (Femara*;* Novartis Pharma Services, Basel, Switzerland) was given as 2.5 mg/day for 1–3 months before HIFU treatment. The dose of letrozole was determined following previous report to relieve clinical symptoms of hypermenorrhea and mass effects of uterine leiomyoma under minimal side effects like hot flush or self-limited muscle soreness (29).

Patients in group C cohorts, accepted monthly short-acting GnRHa including leuprolide acetate (Lupron Depot 3.75 mg; Takeda Pharma, Osaka, Japan) or triptorelin (Diphereline P.R. 3.75 mg; Ipsen Pharma, Paris, France) based on physician's preference. Uterine leiomyoma with iso-intensity or hyper-intensity signal in the MRI scan was the major factor for premedication. A total 1 to 3 dosages of GnRHa were given according to patient's clinical symptom, side effects or other situations.

Patients receiving GnRHa or letrozole premedication had monthly surveillance of the clinical symptoms and uterine leiomyoma status. The volume change of the leiomyomas in both group B and C were evaluated under ultrasound. All of the possible adverse events were recorded on every visit of clinics.

### The pretreatment evaluation of HIFU

The pretreatment evaluation of HIFU included basic medical history, physical examination, clinical symptoms, gynecologic ultrasound and pelvic (or pelvo-abdominal) magnetic resonance imaging (MRI). The current study excluded those with obvious contraindications for HIFU treatment, such as severe abdominal wall scar or extremely retroverted uterus with bowel interference of acoustic channel. MRI included T2-weighted and T1 contrast enhanced images to demonstrate the vascularity of uterine leiomyoma from signal intensity that can predict the ablation response of HIFU treatment (30). To differentiate leiomyosarcoma from leiomyoma, reading of MRI combined diffusion weighted imaging (DWI) and apparent diffusion coefficient (ADC) (31).

### The HIFU treatment and post-treatment surveillance

HIFU treatment of uterine leiomyoma was performed with the ultrasound-guided HIFU machine (JC; Chongqing Haifu Medical Technology, Chongqin, China). Based on the manufacturer's protocol and treatment procedures, the targeted treatment area was confirmed under the guidance of HIFU ultrasound. Then the energy focus was placed inside the treatment area after we make sure the safety margin around the leiomyoma, and the ablation energy was transmitted. When the visible coagulation necrosis was generated on the ultrasound during HIFU treatment, we furtherly inputted energy layer by layer to extended the echogenic necrotic area to the entire uterine fibroids. HIFU treatment was then finished after the enough ablation was confirmed by ultrasound. After HIFU ablation, all patients received post-HIFU MRI study on the next day to see the non-perfusion volume (NPV) ratio as an immediate evaluation of response of ablation. All associated adverse events within 30 days after HIFU ablation were recorded. The safety and toxicity were accessed with the classification system of society of interventional radiology (SIR), including minor or major complications (32). All the patients were suggested to a follow-up schedule of every 3–6 months in the first year and 6–12 months thereafter. Gynecologic ultrasound examination for the treated leiomyoma was included in each clinic visit.

### The HIFU treatment profile

Based on previous study, the volume of leiomyoma was calculated as following equation: *V* = 0.5233 x length x width x depth (33). The volume reduction rate was calculated as 1 minus the changing ratio, and the changing ratio was calculated with the post-HIFU leiomyoma volume divided by its original volume. The sonication time was HIFU energy emission time alone, and the treatment time was the total duration from treatment start until end of treatment. We also calculated other parameters of HIFU treatment efficiency including “energy efficiency (EEF) = energy/volume (J/cm^3^)”, “treatment speed = volume/treatment duration (cm^3^/h)”, and “power rate = energy/treatment duration (J/h)”. The three index markers of NPV generation efficiency included “treatment efficiency (TEF) = NPV/treatment duration (cm^3^/min)”, “NPV per sonication = NPV/sonication time (cm^3^/min)”, and “NPV per energy = NPV/energy (cm^3^/J)”.

### Statistical analysis

Baseline comparison of patient characteristics in different groups cohorts were performed with paired student *t*-test to demonstrate the difference in these groups. After HIFU treatment, the related parameters of HIFU profile and the obtained volume reduction rates at different surveillance point over time calculated and compared in different patient groups by the Chi-Squared test, paired *t*-test, and ANOVA analysis. The analysis was considered to be significant when the *p*-value was < 0.05. All above calculation was done by SPSS (IBM, version 22).

## Results

### Patients characteristics

From January 2018 to April 2021, 39 patients with single uterine leiomyoma treated with HIFU ablation at Chang Gung Memorial Hospital of Linkou branch were collected for analysis. Patients were divided into 3 groups according to pre-HIFU medication, group A without medication (*N* = 21), group B using letrozole (*N* = 7), and group C accepting GnRHa (*N* = 11) (Table 1). Among the 3 groups, patients of group C had higher median age than those of group A with statistical significance (47.0 vs. 40.9 years, *p* = 0.029), but no obvious difference between median age of patients of group B and C (43.2 vs. 47.0, *p* = 0.669). The pre-HIFU symptoms including hypermenorrhea, dysmenorrhea, compression symptoms such as voiding frequency or abdominal fullness found in 22 patients (56.4%), 7 patients (17.9%), and 20 patients (51.3%) without differences among the three groups (*p* = 0.332). Two cases in group A had no symptoms and received HIFU ablation for conception and infertility issues. The body mass index (BMI), skin thickness, focus depth, abdominal operation history, parity, retroverted/anteverted uterus, or leiomyoma location among these 3 groups were not significantly different between patients of 3 groups. Patients of group C had a trend of higher intensity signal of T2 MRI view than those of group A and group B (45.5 vs. 9.5 vs. 14.3%, *p* = 0.055). There was no significant difference in the T1 contrast enhanced MRI view (*p* = 0.401) and the size of leiomyomas between patients of 3 groups (A: 251.4, B: 360.6, C: 409.4 cm^3^, *p* = 0.250).

**Table 1:** Patient characteristics.

	**Nil (*N =* 21)**	**Letrozole (*N =* 7)**	**GnRHa (*N =* 11)**	***p*-value**
Age (yrs), median (range)	40.9 (27.4–51.8)	43.2 (39.1–48.6)	47.0 (33.6–54.4)	0.033 N vs. L: 0.386 N vs. G: 0.029 L vs. G: 0.669
BMI, median (range)	21.4 (16.8–33.6)	20.2 (18.7–21.1)	22.2 (19.8–24.8)	0.286 N vs. L: 0.258 N vs. G: 0.962 L vs. G: 0.439
Skin thickness, median cm (range)	1.47 (0.63–4.0)	0.88 (0.7–2.0)	1.38 (0.50–2.10)	0.140 N vs. L: 0.173 N vs. G: 0.351 L vs. G: 0.832
Focus depth median cm (range)	9.31 (7.25–12.17)	8.72 (8.01–11.42)	10.07 (6.89–13.62)	0.503 N vs. L: 0.802 N vs. G: 0.709 L vs. G: 0.485
Mass volume measured on HIFU day, median cm^3^ (range)	251.4 (34.7–783.6)	360.6 (172.9–816.6)	409.4 (145.4–831.2)	0.250 N vs. L: 0.663 N vs. G: 0.239 L vs. G: 0.879
**Abdominal OP history**, ***N*** **(%)**
Yes	6 (28.6)	0	4 (36.4)	0.205
No	15 (71.4)	7 (100)	7 (63.6)	
**Parity**, ***N*** **(%)**
Nulliparity	12 (57.1)	3 (42.9)	4 (36.4)	0.156
Primiparity	5 (23.8)	0	1 (9.1)	
Multiparity	4 (19.0)	4 (57.1)	6 (54.5)	
**Initial symptoms**, ***N*** **(%)**^**a**^
Hypermenorrhea	12 (57.1)	6 (85.7)	4 (36.4)	0.332
Dysmenorrhea	3 (14.3)	2 (28.6)	2 (18.2)	
Compression symptoms	9 (42.9)	2 (28.6)	9 (81.8)	
For pregnancy	2 (9.5)	0	0	
**RVF or AVF**, ***N*** **(%)**^**b**^
AVF uterus	15 (78.9)	5 (83.3)	9 (81.8)	0.965
RVF uterus	4 (21.1)	1 (16.7)	2 (18.2)	
**Mass location**, ***N*** **(%)**
Fundus or lateral	4 (19.0)	2 (28.6)	2 (18.2)	0.924
Anterior wall	10 (47.7)	3 (42.8)	4 (36.4)	
Posterior wall	7 (33.3)	2 (28.6)	5 (45.4)	
**MRI T2 intensity**, ***N*** **(%)**
High intensity	2 (9.5)	1 (14.3)	5 (45.5)	0.055
Low intensity	19 (90.5)	6 (85.7)	5 (45.5)	
Mixed intensity	0	0	1 (9.1)	
**MRI T1-C+** **intensity**, ***N*** **(%)**
High intensity	1 (4.8)	0	0	0.401
Iso-intensity	3 (14.3)	1 (14.3)	5 (45.5)	
Low intensity	16 (76.2)	6 (85.7)	5 (45.5)	
Mixed intensity	1 (4.8)	0	1 (9.1)	

GnRHa, gonadotrophin releasing hormone analog; BMI, body mass index; HIFU, high intensity focused ultrasound; OP, operation; RVF, retroflexed uterus; AVF, anteflexed uterus; N, nil; L, Letrozole; G, GnRHa.

^a^May have multiple symptoms.

^b^Missing data in 3 patients.

### Response of pre-HIFU medication

The volume change before and after studied medication including letrozole and GnRHa in group B and C patients respectively were demonstrated in Table 2. Two patients (28.6%) used letrozole for 1 month, and 5 patients (71.4%) for 3 months. The number of patients used 1 month, 2 months, and 3 months of GnRHa were 3 (27.3%), 7 (63.6%) and 1 (9.1%), respectively. The GnRHa used for pre-HIFU treatment in group C patients can generate significantly more volume reduction of leiomyomas than letrozole used in group B patients (38.6 vs. 16.4%, *p* = 0.007). Besides, no obvious discomforts were found in group B patients during pre-HIFU medication, and three of group C patients (27.3%) had hypoestrogenic symptoms including hot flush or night sweating (*p* = 0.170).

**Table 2:** Pre-HIFU medical treatment response.

	**Letrozole group (*N =* 7)**	**GnRHa group (*N =* 11)**	***p*-value**
Treatment details	1 months: 2	1 months (3.75 mg): 3	–
	2 months: 0	2 months (3.75 mg): 7	
	3 months: 5	3 months: 1^a^	
Volume before medication (cm^3^)	438.7 (242.8–1,004.7)	652.3 (209.3–1,096.1)	0.123
Volume after medication (cm^3^)	360.6 (172.9–816.6)	409.4 (145.4–831.2)	0.657
Volume reduction rates (%)	16.4 (−6.3–37.8)	38.6 (18.5–60.2)	0.007
Hypoestrogenic symptoms, *N* (%)	0	3 (27.3)	0.170

GnRHa, gonadotrophin releasing hormone analog; HIFU, high intensity focused ultrasound.

a 3 doses of 3.75 mg or 1 dose of 11.25 mg.

### HIFU treatment profiles among 3 groups

The treatment profiles were demonstrated in Table 3. Compared with patients of A and B, those of Group C had longer sonication time (A vs. C, 739 vs. 1,137 s, *p* = 0.001; B vs. C, 711 vs. 1,137 s, *p* = 0.009), longer treatment duration (A vs. C, 95 vs. 148 min, *p* = 0.003; B vs. C, 94 vs. 148 min, *p* = 0.020), and higher ablation energy (A vs. C, 293,911 vs. 452,572 Joule, *p* = 0.002; B vs. C, 282,639 vs. 452,572 Joule, *p* = 0.010). The NPV ratio (*p* = 0.231), energy efficiency (*p* = 0.425), treatment speed (*p* = 0.273), and power rates (*p* = 0.863) were not different significantly among the 3 groups. Patients of group B had a trend of higher treatment efficiency than those of group A and C (A vs. B, 2.39 vs. 4.52 cm^3^/min, *p* = 0.053; C vs. B, 2.34 vs. 4.52 cm^3^/min, *p* = 0.078).

**Table 3:** HIFU treatment profile.

	**Nil (*N =* 21)**	**Letrozole (*N =* 7)**	**GnRHa (*N =* 11)**	***p*-value**
Sonication time (s) Median (range)	739 (320–1,226)	711 (345–887)	1,137 (777–1,606)	0.001 N vs. L: 0.972 N vs. G: 0.001 L vs. G: 0.009
Treatment duration (min) Median (range)	95 (47–158)	94 (46–174)	148 (96–237)	0.002 N vs. L: 0.997 N vs. G: 0.003 L vs. G: 0.020
Energy (J) Median (range)	293,911 (127,850–490,100)	282,639 (137,550–354,500)	452,572 (310,350–642,100)	0.001 N vs. L: 0.971 N vs. G: 0.002 L vs. G: 0.010
Non-perfusion volume ratio (NPV, %), median (range)	88.5 (61–100)	96 (76–100)	87.7 (72–98)	0.231 N vs. L: 0.258 N vs. G: 0.978 L vs. G: 0.259
Energy efficiency (E/V) (J/cm^3^), median (range)	1,775.1 (424.5–5,609.1)	1,010.4 (305.8–1,772.7)	1,552.7 (404.8–4,123.7)	0.425 N vs. L: 0.392 N vs. G: 0.894 L vs. G: 0.677
Treatment speed (cm^3^/h) Median (range)	187.8 (27.6–522.4)	292.7 (59.6–612.4)	187.9 (44.5–484.2)	0.273 N vs. L: 0.272 N vs. G: 1.000 L vs. G: 0.344
Power rate (J/h) Median (range)	186,249.2 (104,604.8–277,933.3)	197,004.7 (105,689.7–271,854.5)	188,733.6 (145,369.9–284,812.5)	0.863 N vs. L: 0.851 N vs. G: 0.988 L vs. G: 0.925
Treat efficiency (NPV/min) Median (range)	2.39 (0.37–5.69)	4.52 (0.89–9.70)	2.34 (0.55–7.05)	0.050 N vs. L: 0.053 N vs. G: 0.997 L vs. G: 0.078
NPV per energy (cm^3^/J)	0.077 (0.039–0.151)	0.074 (0.054–0.140)	0.046 (0.030–0.063)	0.012 N vs. L: 0.947 N vs. G: 0.010 L vs. G: 0.101
NPV per sonication (cm^3^/s)	30.96 (15.94–60.22)	29.52 (21.08–55.84)	18.09 (12.13–25.17)	0.12 N vs. L: 0.953 N vs. G: 0.010 L vs. G: 0.098

GnRHa, gonadotrophin releasing hormone analog; HIFU, high intensity focused ultrasound; NPV, non-perfusion volume.

**Figure 1:**
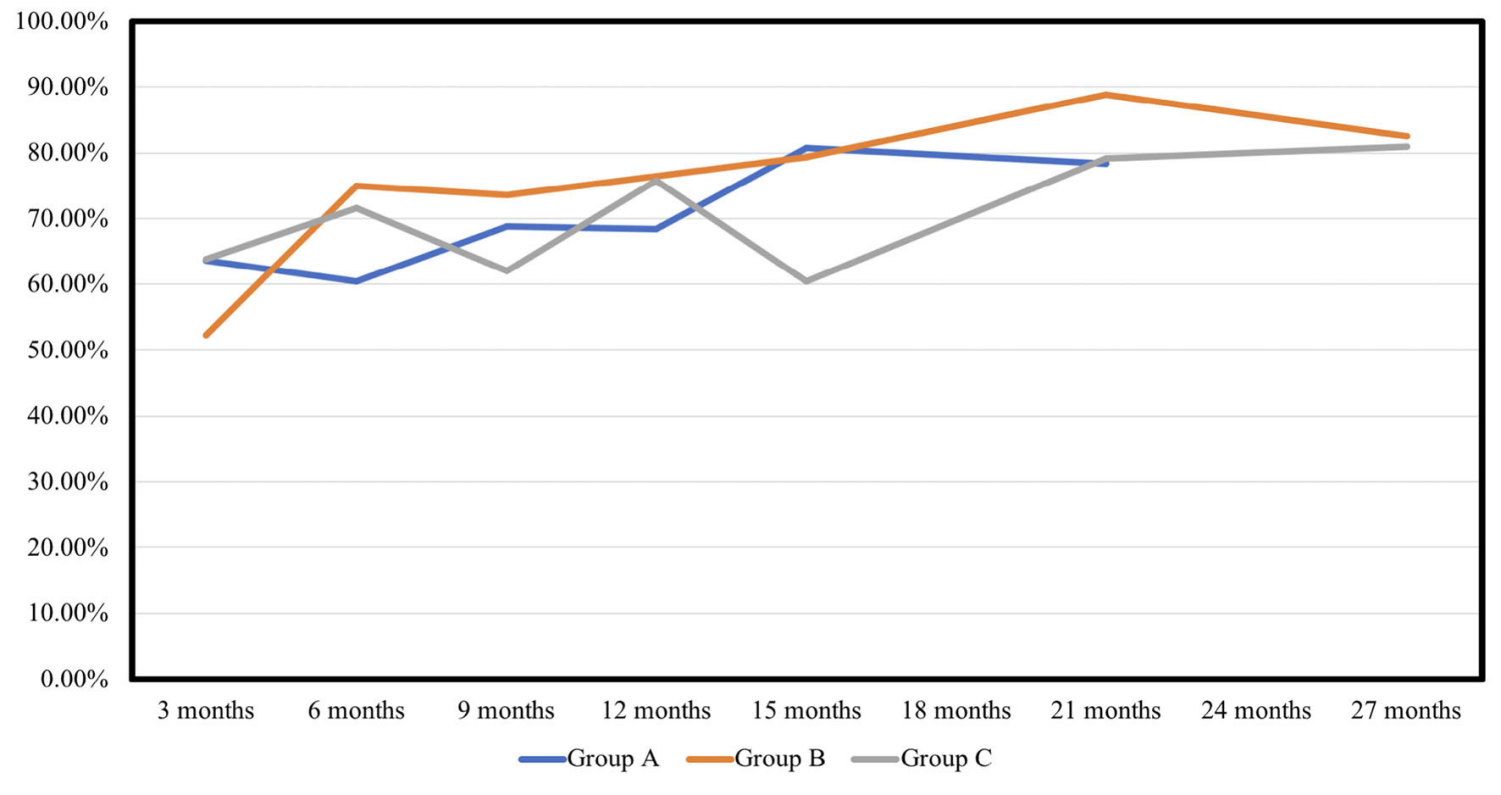
Volume reduction rates of fibroid.

The NPV per sonication of group B was similar to that of group A (A vs. B, 30.96 vs. 29.52 cm^3^/s, *p* = 0.953), and higher than that of group C in group (C vs. B, 18.09 vs. 29.52 cm^3^/min, *p* = 0.098). The NPV per energy had similar results in the 3 groups (A vs. B, 0.077 vs. 0.074 cm^3^/J, *p* = 0.947; C vs. B, 0.046 vs. 0.074 cm^3^/J, *p* = 0.101). As shown in Table 4 and Figure 1 showed the volume reduction rates had no significant difference at the follow-up times in the 3 groups.

**Table 4:** Volume reduction rates of uterine fibroid after HIFU treatment at different follow-up timing.

	**Nil (*N =* 21)**	**Letrozole (*N =* 7)**	**GnRHa (*N =* 11)**	***p*-value**
3 months (%)	*N =* 16 54.5 (10.5–83.4)	*N =* 7 52.1 (6.9–78.7)	*N =* 9 63.8 (45.4–85.9)	0.448 N vs. L: 0.965 N vs. G: 0.519 L vs. G: 0.497
6 months (%)	*N =* 10 60.8 (3.3–87.9)	*N =* 2 74.9 (65.5–84.4)	*N =* 5 71.6 (44.2–87.4)	0.646 N vs. L: 0.758 N vs. G: 0.724 L vs. G: 0.987
9 months (%)	*N =* 10 58.7 (15.1–82.1)	*N =* 3 73.6 (65.6–81.7)	*N =* 2 62.1 (60.9–63.3)	0.445 N vs. L: 0.413 N vs. G: 0.965 L vs. G: 0.749
12 months (%)	*N =* 4 68.6 (65.3–70.4)	*N =* 0	*N =* 2 75.7 (68.2–83.2)	0.220
15 months (%)	*N =* 2 80.7 (75.7–85.7)	*N =* 2 79.4 (72.9–85.9)	*N =* 1 60.5	0.304
18 months (%)	*N =* 0	*N =* 0	*N =* 0	NA
21 months (%)	*N =* 3 58.7 (19.3–83.1)	*N =* 2 88.9 (83.6–94.2)	*N =* 1 79.2	0.559
24 months (%)	*N =* 0	*N =* 0	*N =* 0	NA
27 months (%)	*N =* 0	*N =* 1 82.6	*N =* 1 81.0	NA

GnRHa, gonadotrophin releasing hormone analog; HIFU, high intensity focused ultrasound.

### HIFU treatment profiles for uterine fibroid over 10 cm

Supplementary Table S1 demonstrated the HIFU parameters profile in patients with leiomyoma size over 10 cm before HIFU treatment among these 3 groups. There were only 2 patients with leiomyoma over 10 cm in group B. Patients of group B had shorter sonication time (A vs. B, 926 vs. 547 s, *p* = 0.088; C vs. B, 1,079 vs. 547 s, *p* = 0.016), shorter treatment duration (A vs. B, 104 vs. 63 min, *p* = 0.174; C vs. B, 135 vs. 63 min, *p* = 0.014), and less ablation energy (A vs. B, 369640 vs. 218225 Joule, *p* = 0.096; B vs. C, 428,260 vs. 218,225 Joule, *p* = 0.019) than those of group A and C. Group B patients had higher energy efficiency by less energy consumption per volume (A vs. B, 882.7 vs. 335.9 J/cm^3^, *p* = 0.100; C vs. B, 966.3 vs. 335.9 J/cm^3^, *p* = 0.056) and faster treatment speed (A vs. B, 279.9 vs. 599.6 cm^3^/h, *p* = 0.013; C vs. B, 234.8 vs. 599.6 cm^3^/h, *p* = 0.006) than that of other 2 groups. The treatment efficiency (A vs. B, 3.63 vs. 8.75 cm^3^/min, *p* = 0.002; C vs. B, 2.88 vs. 8.75 cm^3^/min, *p* = 0.001), NPV per sonication (A vs. B, 21.97 vs. 40.45 cm^3^/s, *p* = 0.011; C vs. B, 18.57 vs. 40.45 cm^3^/s, *p* = 0.003), and NPV per energy (A vs. B, 0.055 vs. 0.101 cm^3^/J, *p* = 0.011; C vs. B, 0.047 vs. 0.101 cm^3^/min, *p* = 0.004) were significantly higher in patients of group B than those of group A and C.

### Adverse events after HIFU treatment

Supplementary Table S2 showed the side effects of medication and HIFU treatment in different groups. There were only minor adverse events in our study including vaginal watery discharge (*N* = 3), vaginal bleeding (*N* = 1), flank soreness (*N* = 1), and low abdominal pain (*N* = 1) that required no treatment. The incidences of the post-HIFU symptoms were not different among these three group patients (*p* = 0.489).

## Discussion

HIFU had already been used in treatment of leiomyoma with non-invasive methods. While compatible with minimal invasive surgery for myomectomy or hysterectomy, HIFU had been already showed the better improvement of quality of life with significance (34). Besides, a prospective “IDEAL” study by Chen et al. (35) also disclosed more rapid improvement of life quality and less morbidity *via* HIFU ablation than surgery. Plenty of articles had shared the experience of size reduction rates of leiomyoma after HIFU ablation. Lee et al. (36) demonstrated reduction rates as 54.7, 62.5, and 73.8% at 3, 6, and 12 months follow-ups after HIFU. The results were consistent between different articles by different groups (37), and also similar with current present study including 50–60%, 60–70%, 70–75%, and 75–80% in 3, 6, 12, and 24 months of surveillance respectively that were demonstrated. Besides, for those fibroids with initial symptoms such as hypermenorrhea, voiding frequency, or abdominal fullness, our study demonstrated there were improved symptoms in all patients after HIFU treatment while the fibroid size was decreased, and no differences were found among three groups.

The mechanism of HIFU ablation had been already well-known. HIFU can eject and collect many ultrasonic beams focusing on the target lesion site, and produce high thermal effects with cavitation effects or mechanical effects leading to coagulation necrosis in the target leiomyomas tissue (38, 39). Besides, the focused hyperthermia produced by HIFU can cause damage of cellular membranes along with protein denaturation, cell energy metabolism disruption after mitochondria dysfunction, and further decreased oxygenation as well as cellular necrosis after vascular destroy (12, 13, 40). In addition, delayed cell apoptosis also appeared after vascular damage and accompanied inflammatory response caused by hyperthermia (12, 41). The necrotic and apoptotic cells after HIFU can be phagocytosed and digested by the inflammatory cells including macrophage (42, 43). Size reduction of leiomyomas after HIFU ablation can be achieved by accumulation of death cell digestion by inflammatory cells as time went on.

Previous literatures had already demonstrated the parameters of HIFU ablation efficiency. Retrospective study by Fan et al. (37) disclosed several factors with significance for energy efficiency of single uterine leiomyoma receiving ultrasound-guided HIFU, and they reported better efficiency in patients with hypodense signal on T2WI of pre-HIFU MRI, large fibroid size, mild enhancement on T1WI of pre-HIFU MRI, and anteverted uterus. Patients with huge size of leiomyoma have higher incidence of tumor-lysis syndrome with hyperuricemia and hyperkalemia after HIFU treatment (44). Therefore, adjuvant medical treatment before HIFU treatment to shrink leiomyoma size and make reinforcement of HIFU efficacy is often used.

Other than reducing size of leiomyoma before HIFU, methods to enhance the treatment efficacy is of interest. Smart et al. (45) reported that a higher energy efficiency by NPV per energy (0.06 vs. 0.03 J/cm^3^, *p* < 0.05) was observed in GnRHa users compared with patients accepting HIFU alone. Yang et al. (18) reported pre-HIFU GnRHa treatment can shorten HIFU treatment duration (102 vs. 49 min, *p* = 0.046), reduce sonication time (25.4 vs. 38.9 min, *p* = 0.048), enhance treatment efficiency (9.9 vs. 23.8 KJ/cm^3^), and increase NPV ratio (69.2 vs. 50.2 %). However, GnRHa frequently causes estrogen-deprived symptoms including hot flush, insomnia vaginal dryness, and loss of bone mineral density (46). Park et al. (47) reported single dose of short-acting GnRHa before HIFU could also enhance energy efficiency (NPV per energy: 0.046 vs. 0.031 cm^3^/J, *p* = 0.041) with less GnRHa-related hypoestrogenic symptoms.

Letrozole is used initially for medical treatment of breast cancer, and the importance was increased since the issues of onco-fertility and fertility assistance after cancer treatment get more and more attention (48). The long-term risky evaluation to neonates and children after the artificial reproductive technologies was needed (49–52), and the consultation from multi-disciplinary collaboration was therefore necessary (53). Comparing with GnRHa, Letrozole also had inferior size reduction effect of leiomyomas. In a randomized controlled clinical trial, the volume reduction of leiomyoma before laparoscopic myomectomy was 45.6% in patients receiving letrozole for 12 weeks and 33.2% in those with short-acting GnRHa for 3 doses (28). The rapid onset of action and avoidance of initial gonadotropin flare with letrozole is an advantage to the patients with symptomatic leiomyoma who are mostly pre-menopausal. Letrozole can inhibit estrogen formation by suppression of aromatase and furtherly decreased the activity of estrogen receptor. From previous research, estrogen receptor had several signaling pathways including epidermal growth factor receptor (EGRF), human epidermal growth factor receptor 2 (HER2, ERBB2), and insulin-like growth factor receptor (IGFR) (54). Letrozole caused regression of tumors of estrogen-dependent human breast cancer MCF-7Ca cells grown in nude mice and increased the number of cells undergoing apoptosis (55). Meresman et al. reported that letrozole enhanced the apoptosis in the endometrial culture of patients with endometriosis (56). Animal study demonstrated apoptosis and cell proliferation in surrounding areas of coagulated necrotic tissues after ablation could be the mechanism of HIFU treatment (12). The study showed that apoptosis index detected by terminal deoxynucleotidyl transferase dUTP nick end labeling (TUNEL) reached a peak value at 72 h after ablation, and the highest proliferating cell nuclear antigen (PCNA)-positive index was found at 144 h after ablation. Apoptotic bodies and oncotic mitochondria in surrounding areas were observed under the electron microscope (12). HIFU ablation can also lead to cell apoptosis and furtherly modulate tumor microenvironment of immunity and the expression of growth factors that can induce antitumor effects (57–59). Therefore, letrozole might enhance the treatment efficacy of HIFU on ablation of uterine leiomyoma.

Although the NPV ratio in our study was no significant differences among groups (*p* = 0.231), patients using letrozole had significantly higher treatment efficiency than those without premedication and those with GnRHa with higher NPV per min (4.52 vs. 2.39 vs. 2.34 cm^3^/min, *p* = 0.050). Comparing with GnRHa patients, letrozole group also had higher NPV per energy (0.074 vs. 0.046 cm^3^/J, *p* = 0.101) and higher NPV per sonication (29.52 vs. 18.09 cm^3^/sonication, *p* = 0.098). The degree of treatment efficacy is ever larger in patients with leiomyoma larger than 10 cm in diameter. Less energy consumption and treatment duration might also result into post-HIFU discomfort and complications. Additionally, only one (9.1%) of our patients receiving pre-HIFU GnRHa had conventional 3 months dosage, but 71.4% of letrozole group cohorts used 3 months regimen, and this may also lead to the difference of efficacy. However, a study of larger sample size should be done in the future to validate our findings.

To the best of our knowledge, the present study is the first one to combine letrozole with HIFU ablation for the treatment of uterine leiomyoma. The limitation of our study was small sample size. Besides, the regimen of GnRHa was also not unified in the medications, dosage, or using duration since it was a retrospective article. Further validation with more patients or a prospective study is needed in the future.

In summary, Letrozole as a pretreatment medication before HIFU therapy of leiomyoma can increase energy efficiency and treatment efficiency of HIFU treatment with less hypoestrogenic symptoms comparing with GnRHa, especially for leiomyoma larger than 10 cm in diameter.

## Data availability statement

The original contributions presented in the study are included in the article/Supplementary material, further inquiries can be directed to the corresponding authors.

## Ethics statement

The studies involving human participants were reviewed and approved by Institutional Review Board of Chang Gung Memorial Hospital (IRB No. 202200674B0, on 9 May 2022). Written informed consent for participation was not required for this study in accordance with the national legislation and the institutional requirements.

## Author contributions

Conceptualization: W-CC, H-HC, and T-CC. Methodology, validation, and data curation: W-CC and C-CH. Software, formal analysis, project administration, funding acquisition, and writing—original draft preparation: W-CC. Investigation: W-CC, C-CH, and T-CC. Resources: H-JH, W-JC, and T-CC. Writing—review and editing: W-CC and H-HC. Visualization: W-CC, C-CH, and H-HC. Supervision: H-HC and T-CC. All authors have read and agreed to the published version of the manuscript.

## Funding

This study was supported by grants from Taiwan's Ministry of Science and Technology (MOST 109-2222-E-182A-002) and Chang Gung Medical Foundation (CORPG3J0441, CRRPG2L0011, CMRPG2L0261).

## Conflict of interest

The authors declare that the research was conducted in the absence of any commercial or financial relationships that could be construed as a potential conflict of interest.

## Publisher's note

All claims expressed in this article are solely those of the authors and do not necessarily represent those of their affiliated organizations, or those of the publisher, the editors and the reviewers. Any product that may be evaluated in this article, or claim that may be made by its manufacturer, is not guaranteed or endorsed by the publisher.
