# Supplementary material for: Letrozole as premedication of high intensity focused ultrasound treatment of uterine fibroids: A retrospective observation study
Source: Front Med (Lausanne). 2022 Dec 6;9:1069654. doi: 10.3389/fmed.2022.1069654 (PMC9763453; doi:10.3389/fmed.2022.1069654)
Supplement: Supplementary file 1 [file Data_Sheet_1.docx]

**Table S1. HIFU treatment profile of uterine fibroid size over 10cm.**

|  | **Nil (N=9)** | **Letrozole (N=2)** | **GnRHa (N=8)** | **p value** |
| --- | --- | --- | --- | --- |
| **Sonication time (seconds)**  **Median (range)** | 926  (593 – 1140) | 547  (345 – 748) | 1079  (873 – 1500) | 0.019  N vs. L: 0.088  N vs. G: 0.329  L vs. G: 0.016 |
| **Treatment duration (mins)**  **Median (range)** | 104  (65 – 146) | 63  (46 – 80) | 135  (96 – 177) | 0.012  N vs. L: 0.174  N vs. G: 0.101  L vs. G: 0.014 |
| **Energy (J)**  **Median (range)** | 369640  (236600 – 455400) | 218225  (137550 – 298900) | 428260  (336480 – 599850) | 0.024  N vs. L: 0.096  N vs. G: 0.369  L vs. G: 0.019 |
| **Non-perfusion volume ratio**  **(NPV, %), median (range)** | 89.7  (61 – 100) | 100 | 91.1  (73 – 98) | 0.460  N vs. L: 0.429  N vs. G: 0.955  L vs. G: 0.538 |
| **Energy efficiency (E/V)**  **(J/cm^3^), median (range)** | 882.7  (424.5 – 1373.3) | 335.9  (305.8 – 366.0) | 966.3  (404.8 – 1568.0) | 0.067  N vs. L: 0.100  N vs. G: 0.851  L vs. G: 0.056 |
| **Treatment speed (cm^3^/hr)**  **Median (range)** | 279.9  (147.9 – 522.4) | 599.6  (586.8 – 612.4) | 234.8  (105.9 – 484.2) | 0.007  N vs. L: 0.013  N vs. G: 0.746  L vs. G: 0.006 |
| **Power rate (J/hr)**  **Median (range)** | 215548.9  (150739.7 – 277933.3) | 201794.0  (179413.0 – 224175.0) | 196513.4  (145369.9 – 284812.5) | 0.623  N vs. L: 0.900  N vs. G: 0.603  L vs. G: 0.985 |
| **Treatment efficiency (NPV/min)**  **Median (range)** | 3.63  (1.81 – 5.69) | 8.75  (7.79 – 9.70) | 2.88  (1.40 – 7.05) | 0.001  N vs. L: 0.002  N vs. G: 0.610  L vs. G: 0.001 |
| **NPV per energy (cm^3^/J)** | 0.055  (0.042 – 0.081) | 0.101  (0.062 – 0.140) | 0.047  (0.032 – 0.057) | 0.005  N vs. L: 0.011  N vs. G: 0.621  L vs. G: 0.004 |
| **NPV per sonication (cm^3^/seconds)** | 21.97  (16.84 – 32.42) | 40.45  (25.06 – 55.84) | 18.57  (12.98 – 22.32) | 0.004  N vs. L: 0.011  N vs. G: 0.591  L vs. G: 0.003 |

GnRHa, gonadotrophin releasing hormone analogue; HIFU, high intensity focused ultrasound; NPV, non-perfusion volume; N, nil; L, Letrozole; G, GnRHa.

**Table S2. Volume reduction rates of uterine fibroid size over 10cm after HIFU treatment at different follow-up timing.**

|  | **Nil (N=9)** | **Letrozole (N=2)** | **GnRHa (N=8)** | **p value** |
| --- | --- | --- | --- | --- |
| **3 months (%)** | N=7  64.4 (38.5 – 82.2) | N=2  46.3 (37.9 – 54.6) | N=7  64.4 (45.4 – 85.9) | 0.341  N vs. L: 0.348  N vs. G: 1.000  L vs. G: 0.349 |
| **6 months (%)** | N=4  71.4 (60.5 – 85.5) | N=0  - | N=5  71.6 (44.2 – 87.4) | 0.981 |
| **9 months (%)** | N=4  71.3 (61.7 – 82.1) | N=1  81.7 | N=2  62.1 (60.9 – 63.3) | 0.215 |
| **12 months (%)** | N=3  68.3 (65.3 – 70.4) | N=0 | N=2  75.7 (68.2 – 83.2) | 0.302 |
| **15 months (%)** | N=1  85.7 | N=1  72.9 | N=1  60.5 | NA |
| **18 months (%)** | N=0 | N=0 | N=0 | NA |
| **21 months (%)** | N=1  83.1 | N=1  83.6 | N=1  79.2 | NA |
| **24 months (%)** | N=0 | N=0 | N=0 | NA |
| **27 months (%)** | N=0 | N=1  82.6 | N=1  81.0 | NA |

GnRHa, gonadotrophin releasing hormone analogue; HIFU, high intensity focused ultrasound; N, nil; L, Letrozole; G, GnRHa.

**Table S3. Adverse events of HIFU treatment.**

| **Group** | **Adverse events, N (%)** | **SIR category** | **p value** |
| --- | --- | --- | --- |
| **Group A (N=21)** | Vaginal bleeding, 1(4.8%)  Vaginal watery discharge, 2(9.5%) | A  A | 0.489 |
| **Group B (N=7)** | Abdominal pain, 1 (14.3%)  Flank soreness, 1(14.3%) | A  A |  |
| **Group C (N=11)** | Vaginal watery discharge, 1 (9.1%) | A |  |

HIFU, high intensity focused ultrasound; SIR, society of interventional radiology.

**Table S4. HIFU treatment profile of uterine fibroid size over 8cm.**

|  | **Nil (N=13)** | **Letrozole (N=7)** | **GnRHa (N=11)** | **p value** |
| --- | --- | --- | --- | --- |
| **Sonication time (seconds)**  **Median (range)** | 839  (423 – 1226) | 711  (345 – 887) | 1137  (777 – 1606) | 0.006  N vs. L: 0.575  N vs. G: 0.030  L vs. G: 0.008 |
| **Treatment duration (mins)**  **Median (range)** | 98  (47 – 158) | 94  (46 – 174) | 148  (96 – 237) | 0.008  N vs. L: 0.981  N vs. G: 0.014  L vs. G: 0.027 |
| **Energy (J)**  **Median (range)** | 334832  (169200 – 490100) | 282639  (137550 – 354500) | 452572  (310350 – 642100) | 0.006  N vs. L: 0.567  N vs. G: 0.034  L vs. G: 0.008 |
| **Non-perfusion volume ratio**  **(NPV, %), median (range)** | 91.2  (61 – 100) | 96.0  (76 – 100) | 87.7  (72 – 98) | 0.254  N vs. L: 0.578  N vs. G: 0.677  L vs. G: 0.225 |
| **Energy efficiency (E/V)**  **(J/cm^3^), median (range)** | 903.1  (424.5 – 1424.7) | 1010.4  (305.8 – 1772.7) | 1552.7  (404.8 – 4123.7) | 0.132  N vs. L: 0.955  N vs. G: 0.128  L vs. G: 0.343 |
| **Treatment speed (cm^3^/hr)**  **Median (range)** | 260.6  (130.6 – 522.4) | 292.7  (59.6 – 612.4) | 187.9  (44.5 – 484.2) | 0.319  N vs. L: 0.894  N vs. G: 0.480  L vs. G: 0.340 |
| **Power rate (J/hr)**  **Median (range)** | 207310.6  (150739.7 – 277933.3) | 197004.7  (105689.7 – 271854.5) | 188733.6  (145369.9 – 284812.5) | 0.563  N vs. L: 0.861  N vs. G: 0.535  L vs. G: 0.913 |
| **Treat efficiency (NPV/min)**  **Median (range)** | 3.29  (1.57 – 5.69) | 4.52  (0.89 – 9.70) | 2.34  (0.55 – 7.05) | 0.103  N vs. L: 0.411  N vs. G: 0.501  L vs. G: 0.086 |
| **NPV per energy (cm^3^/J)** | 0.066  (0.039 – 0.113) | 0.074  (0.054 – 0.140) | 0.045  (0.030 – 0.063) | 0.034  N vs. L: 0.741  N vs. G: 0.100  L vs. G: 0.044 |
| **NPV per sonication (cm^3^/seconds)** | 26.35  (15.94 – 45.36) | 29.52  (25.08 – 55.84) | 18.08  (12.14 – 25.17) | 0.033  N vs. L: 0.749  N vs. G: 0.094  L vs. G: 0.043 |

GnRHa, gonadotrophin releasing hormone analogue; HIFU, high intensity focused ultrasound; NPV, non-perfusion volume; N, nil; L, Letrozole; G, GnRHa.

**Table S5. Volume reduction rates of uterine fibroid size over 8cm after HIFU treatment at different follow-up timing.**

|  | **Nil (N=13)** | **Letrozole (N=7)** | **GnRHa (N=11)** | **p value** |
| --- | --- | --- | --- | --- |
| **3 months (%)** | N=10  63.7 (38.5 – 83.4) | N=7  52.2 (6.9 – 78.7) | N=9  63.8 (45.4 – 85.9) | 0.354  N vs. L: 0.400  N vs. G: 1.000  L vs. G: 0.411 |
| **6 months (%)** | N=6  60.5 (3.3 – 85.5) | N=2  74.9 (65.5 – 84.4) | N=5  71.6 (44.2 – 87.4) | 0.664  N vs. L: 0.745  N vs. G: 0.729  L vs. G: 0.985 |
| **9 months (%)** | N=7  68.7 (54.5 – 82.1) | N=3  73.6 (65.6 – 81.7) | N=2  62.1 (60.9 – 63.3) | 0.343  N vs. L: 0.663  N vs. G: 0.591  L vs. G: 0.314 |
| **12 months (%)** | N=3  68.3 (65.3 – 70.4) | N=0  - | N=2  75.7 (68.2 – 83.2) | 0.302 |
| **15 months (%)** | N=2  80.7 (75.7 – 85.7) | N=2  79.4 (72.9 – 85.9) | N=1  60.5 | 0.304 |
| **18 months (%)** | N=0 | N=0 | N=0 | NA |
| **21 months (%)** | N=2  78.4 (73.8 – 83.1) | N=2  88.9 (83.6 – 94.2) | N=1  79.2 | 0.443 |
| **24 months (%)** | N=0 | N=0 | N=0 | NA |
| **27 months (%)** | N=0 | N=1  82.6 | N=1  81.0 | NA |

GnRHa, gonadotrophin releasing hormone analogue; HIFU, high intensity focused ultrasound; N, nil; L, Letrozole; G, GnRHa.
